# Quality of life in hospitalized COVID-19 patients: the role of psychosocial, inflammatory, and dopaminergic pathways

**DOI:** 10.3389/fpsyg.2025.1684510

**Published:** 2026-01-07

**Authors:** M. Graça Pereira, Margarida Vilaça, Martim Santos, Sandra Carvalho, Jorge Leite, André Santos Silva, Marco Cosentino, Franca Marino, Massimiliano Legnaro, Fernanda Leite

**Affiliations:** 1 Psychology Research Centre (CIPsi), School of Psychology, University of Minho, Braga, Portugal; 2 Psychological Neuroscience Laboratory, Psychology Research Center (CIPsi), School of Psychology, University of Minho, Braga, Portugal; 3 Basic Psychology Department, School of Psychology, University of Minho, Braga, Portugal; 4 RISE-Health, CINTESIS.UPT, Portucalense University, Porto, Portugal; 5 Infectious Diseases Department, Santo António University Hospital Center, Porto, Portugal; 6 Abel Salazar Biomedical Sciences Institute, University of Porto, Porto, Portugal; 7 Center of Research in Medical Pharmacology, University of Insubria, Varese, Italy; 8 Institute for Research and Innovation in Health (i3S), University of Porto, Porto, Portugal; 9 Department of Transfusion Medicine, Santo António University Hospital Center, Porto, Portugal; 10 Public Health and Forensic Sciences, and Medical Education Department, Faculty of Medicine, University of Porto, Porto, Portugal

**Keywords:** COVID-19 patients, posttraumatic stress, rumination, inflammation, dopaminergic expression, psychological morbidity, quality of life

## Abstract

**Introduction:**

A significant proportion of COVID-19 survivors continues to suffer from persistent physical and psychological sequelae after hospital discharge.

**Materials and methods:**

This cross-sectional study aimed to analyze the roles of psychosocial, inflammatory, and dopaminergic pathways in the relationship between posttraumatic stress symptoms (PTSS), rumination, and quality of life (QoL) in patients hospitalized with COVID-19, as well as the moderating role of time since discharge. A total of 207 participants were divided into two groups based on their discharge timing: those discharged 24 months prior (cohort I) and those discharged 6 to 12 months prior (cohort II). Data were collected at a single time point using validated measures of PTSS, rumination, psychological morbidity (anxiety and depression), loneliness, satisfaction with life, posttraumatic growth (PTG), and QoL.

**Results:**

PTSS was positively associated with psychological morbidity and the expression of dopaminergic receptor (DR) D1 in peripheral blood mononuclear cells (PBMCs). Rumination was positively and strongly associated with PTG but negatively and weakly associated with satisfaction with life. Psychological morbidity was positively linked to loneliness and negatively associated with DRD1 expression in PBMCs and with physical and mental QoL. Several significant pathways were observed, particularly between PTSS and both QoL dimensions. The moderating role of time since discharge revealed significant differences, suggesting that psychosocial, inflammatory, and dopaminergic dynamics are more pronounced in patients from cohort II.

**Discussion:**

This study underscores the complex interplay of psychosocial and neurobiological processes associated with long-term QoL, highlighting the need for a prompt biopsychosocial care approach to optimize recovery and health outcomes following COVID-19 infection.

## 1 Introduction

Since its emergence in late 2019, the novel severe acute respiratory syndrome coronavirus (SARS-CoV-2) has caused more than 770 million confirmed COVID-19 cases and more than 7 million associated deaths across 234 countries (World Health Organization, 2024). In addition, the rapid global spread of SARS-CoV-2 led to a substantial increase in hospitalizations worldwide, with a global peak during the autumn and winter. According to a nationwide study, COVID-19 hospitalizations peaked at approximately 34,697 patients on the 23rd of November 2020, while intensive care unit (ICU) admissions peaked at 3,848 patients on the 25th of November 2020 (Roncati et al., 2024).

Hospitalization is generally a stressful experience (Alzahrani, 2021). During the early stages of the COVID-19 pandemic, it was further exacerbated by strict isolation measures, family visitor restrictions, and limited contact with healthcare professionals, resulting in significant psychological consequences for patients (Evans et al., 2021). Studies on patients discharged from hospitals have consistently reported significantly lower quality of life (QoL) among those who were hospitalized compared to non-hospitalized individuals, likely due to persistent symptoms associated with the post-acute sequelae of SARS-CoV-2 infection (Evans et al., 2021; Garrigues et al., 2020; Huang et al., 2021; Vlake et al., 2021). Moreover, prior COVID-19 infection has been associated with an increased risk of mental health problems several months after the acute phase, underscoring the relevance of assessing psychosocial sequelae in post-COVID populations (Penninx et al., 2022). The most common psychological symptoms reported by COVID-19 survivors include psychological morbidity (i.e., anxiety and depressive symptoms) and posttraumatic stress symptoms (PTSS; National Institute for Health and Care Excellence, 2020; Thye et al., 2022), with meta-analytic findings estimating prevalence rates between 21 and 31% (Khraisat et al., 2022; Pitron et al., 2022). Although satisfaction with life has not yet been investigated among hospitalized COVID-19 patients, a significant decline in satisfaction with life following COVID-19 infection has been reported (Thompson et al., 2022), highlighting the need for targeted research in this population.

Ruminative thoughts about the hospitalization experience (e.g., repetitive distressing memories) may persist long after physical recovery, exacerbating PTSS, anxiety, and depression (Di Tella and Romeo, 2025; Sun et al., 2021). As a result, prolonged rumination has been associated with mental health symptoms and an adverse impact on long-term QoL among COVID-19 survivors (Juczyński et al., 2023; Vlake et al., 2021), underscoring rumination’s central role in shaping mental and functional recovery in the post-COVID period. Rumination also has physiological consequences that predict inflammation (Szabo et al., 2022). In addition, feelings of loneliness, a typical result of prolonged hospitalization, quarantine restrictions, and post-illness stigma, have also been negatively associated with QoL in discharged patients, with hospitalized patients reporting greater loneliness and lower QoL compared to non-hospitalized patients (Sipowicz et al., 2023).

Traumatic and stressful events, such as hospital admission due to COVID-19, can lead to psychological growth, enhancing both physical and mental QoL (Sun et al., 2021). Posttraumatic growth (PTG) is defined as positive psychological changes and improvements (e.g., personal, interpersonal, and spiritual changes) that occur following a traumatic event (Tedeschi and Calhoun, 2004). Although few studies have focused on PTG in COVID-19 survivors, emerging research suggests that many patients experience positive psychological changes after overcoming acute COVID-19 infection (Özgüç et al., 2022; Sun et al., 2021).

Beyond the mental and physical health impacts, COVID-19 is characterized by significant systemic inflammation, particularly in patients with severe disease progression (Hariyanto et al., 2021). Altered levels of inflammatory markers, such as C-reactive protein (CRP), ferritin, fibrinogen, and the neutrophil-to-lymphocyte ratio (NLR), are frequently observed in hospitalized COVID-19 patients (Kalaiselvan et al., 2023; Liu et al., 2020). The inflammatory response may negatively affect patients’ QoL, contributing to persistent physical symptoms, psychological morbidity, and functional impairment during recovery (Ceban et al., 2022; Maamar et al., 2022). Additionally, the dysregulation of the dopaminergic system, observed in the context of viral infections and systemic inflammation, may impair immune regulation and neurobehavioral functions, further compromising QoL (Eidhof et al., 2024; Rasmi et al., 2024). Although peripheral dopaminergic receptor (DR) expression cannot be directly interpreted as a measure of central dopaminergic functioning, immune cells do express dopamine (DA) receptors and related signaling components, supporting a relevant neuroimmune pathway (McKenna et al., 2002; Pacheco et al., 2014). Recent evidence also suggests that SARS-CoV-2 may affect dopaminergic signaling, making peripheral DR expression a biologically meaningful, albeit indirect, marker of post-COVID-19 neuroimmune alterations (Limanaqi et al., 2022; Tokano et al., 2022).

Several studies have addressed the relationship between PTSS, rumination, psychological deterioration, and increased inflammation, ultimately impairing QoL (e.g., Kachadourian et al., 2021; Michopoulos et al., 2017; Szabo et al., 2022). Prior research has also explored the mediating role of psychological variables, such as anxiety and depression (Kim et al., 2018), loneliness (Gabarrell-Pascuet et al., 2023), PTG (Li et al., 2025), and satisfaction with life (Martins et al., 2022), in individuals exposed to traumatic events. Similarly, the mediating effect of inflammatory markers has been reported in studies focused on traumatic experiences (Zagaria et al., 2024) and QoL (e.g., Nowakowski et al., 2016). However, while the relationships between PTSS, including rumination, and psychological and inflammatory dysregulation are well established, how psychological symptoms and biological markers jointly shape long-term outcomes after COVID-19 hospitalization remains unclear. In particular, the role of the dopaminergic system, which is known to regulate stress responses, emotional processing, and cognitive control, remains underexplored in COVID-19 survivors. Finally, the persistence of COVID-19 health consequences after discharge is not consensual, with some cohort studies suggesting that symptom burden decreases over time (e.g., Yang et al., 2022), while others report that a substantial proportion of patients continue to experience long-term symptoms even years after discharge (Rahmati et al., 2025). Addressing this gap is crucial for informing targeted clinical and rehabilitation interventions.

The present study was conceptually based on the biobehavioral model of negative emotionality (Renna, 2021), which provides a comprehensive framework for understanding how negative emotionality contributes to inflammation and, subsequently, to poor long-term health outcomes. According to this model, negative emotions (e.g., anxiety, PTSS) promote immune system activation and/or dysfunction through a cascade of psychosocial and physiological processes, thereby increasing the risk of morbidity and mortality. In this context, emotional regulation plays a critical role in the relationship between emotion-related neural activation and inflammation.

**Figure 1:**
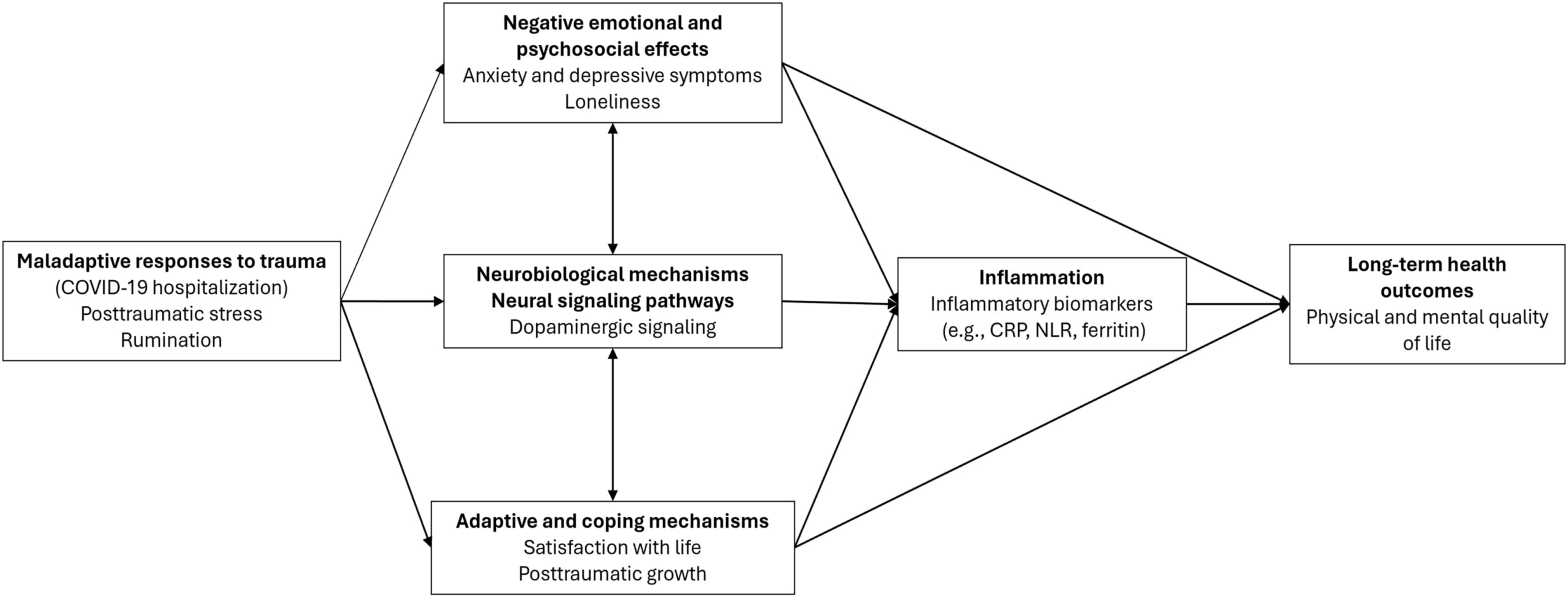
Proposed theoretical model adapted from the biobehavioral model of negative emotionality and the literature review.

Building upon this model, it is imperative to broaden the theoretical framework by integrating neurobiological mechanisms (e.g., dopaminergic signaling) that may interact with psychosocial factors to modulate QoL. Integrating this component is particularly pertinent for individuals exposed to traumatic events, such as hospitalization due to COVID-19, where psychological distress and biological dysregulation may jointly determine long-term recovery outcomes. While neuroinflammatory processes (Yang and Jiang, 2020; Yontar and Mutlu, 2024) and dopaminergic markers (Liu M. N. et al., 2023; Torrisi et al., 2019) have been implicated in the pathophysiology of posttraumatic stress and linked to various health outcomes, these neurobiological factors have not yet been integrated into models explaining how these mechanisms shape QoL. Specifically, the role of neurobiological factors in psychological constructs such as PTG (Glazebrook et al., 2023), satisfaction with life (Hamer and Chida, 2011; Uchino et al., 2018), loneliness (Zilioli and Jiang, 2021), and psychological morbidity (Harsanyi et al., 2023; Hassamal, 2023) remains unexplored within the context of the associations between PTSS, rumination, and QoL. By integrating such psychosocial variables, inflammatory markers (CRP, ferritin, NLR), and the expression of the five DRs (DRD1 to DRD5) in PBMCs into a theoretically proposed model (Figure 1), this study also takes an exploratory approach beyond the original theoretical framework to provide a more comprehensive understanding of the biopsychosocial pathways associated with QoL in patients hospitalized with COVID-19. We hypothesize that psychological factors, inflammatory biomarkers, and DR expression will have an indirect effect on the negative relationship between PTSS/rumination and QoL (Hypothesis 1) and that time since hospital discharge will moderate the proposed theoretical model (Hypothesis 2).

## 2 Materials and methods

### 2.1 Sample and procedure

**Figure 2:**
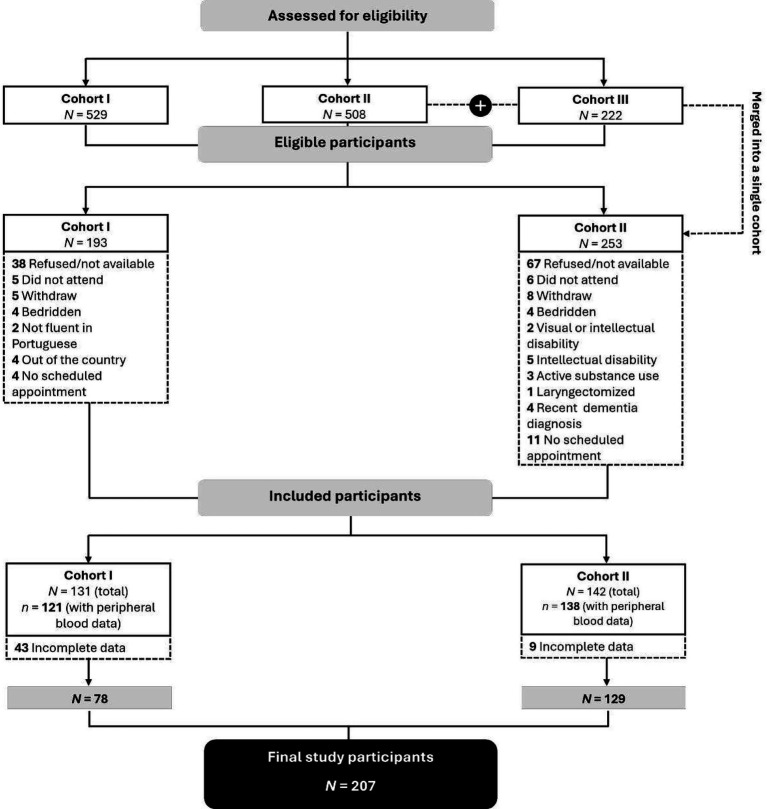
CONSORT flow diagram.

This cross-sectional observational study is part of a larger cohort study examining the effects of psychological factors, inflammatory markers, and dopaminergic pathways on cognitive function and QoL in patients hospitalized with COVID-19. Patients discharged during the typical peak periods of the pandemic, i.e., from October to March 2020/2021 (cohort I), October to March 2021/2022 (cohort II), and October to March 2022/2023 (cohort III), were considered for inclusion. Due to a significant reduction in COVID-19 hospitalizations in Portugal after the winter of 2021, the last two periods were combined, resulting in two analytical discharge-time cohorts: October to March 2020/2021 (cohort I) and October 2021 to March 2023 (cohort II) (Figure 2).

Data were collected at a single time point at a central hospital in northern Portugal, between January 2023 and March 2024. Inclusion criteria were as follows: (i) age between 18 and 75 years; (ii) diagnosis of COVID-19 as the primary or secondary reason for hospitalization; (iii) hospitalization during one of the two cohort periods; and (iv) follow-up at the outpatient infectious diseases clinic. Individuals were excluded if any of the following criteria were met: (a) documented psychiatric disorders, dementia, or a history of brain injury; (b) receipt of chemotherapy or radiotherapy during the data collection period; or (c) long-term residency in a skilled nursing facility.

All eligible patients were consecutively invited by their physicians to participate during routine visits to the hospital’s outpatient infectious diseases clinic. All participants provided written informed consent before participation and were informed of their right to withdraw at any time without consequences. Personal data were anonymized and handled confidentially in accordance with local data protection regulations. SARS-CoV-2 infection was confirmed using qualitative results (positive or negative) from real-time reverse transcriptase polymerase chain reaction (RT-qPCR). After completing the questionnaires, peripheral blood was collected on-site at the hospital to assess inflammatory markers. PBMC samples were then isolated and cryopreserved until DR expression was determined.

The final sample comprises 207 participants who completed the questionnaires and collected peripheral blood samples. A summary of participant recruitment is provided in Figure 2.

This study was approved by the Ethics Committee of the central hospital where data collection took place [Ref. 2022.069(054-DEFI/055-CE)] and followed the principles of the Declaration of Helsinki.

### 2.2 Measures

#### 2.2.1 Sociodemographic and clinical questionnaire

The sociodemographic and clinical questionnaire gathers information on sociodemographic characteristics (e.g., sex, age, marital status) and clinical characteristics (e.g., hospitalization duration, time since discharge, COVID-19 severity) of patients. COVID-19 severity was assessed by the outpatient infectious diseases clinic physician, using the National Institute of Allergy and Infectious Diseases Ordinal Scale (NIAID-OS). This 8-point ordinal scale ranges from 1 (not hospitalized with no limitation of activities) to 8 (death). In this study, only categories 3 (hospitalized, not requiring supplemental oxygen—not in need of ongoing medical care) to 7 (hospitalized, on medical ventilation or extracorporeal membrane oxygenation [ECMO]) were employed to assess participants’ COVID-19 severity.

#### 2.2.2 Short-form health survey (SF-12)

SF-12 (Ferreira et al., 2012) includes 12 items to assess health-related QoL through eight health dimensions: physical function, physical performance, bodily pain, general health perceptions, vitality, social function, emotional performance, and mental health. SF-12 yields two summary measures, the physical (PSM) and mental (MSM), with higher scores indicating better health-related QoL. Items are rated using three- and five-point Likert scales. Cronbach’s alpha coefficients for the Portuguese version were 0.86 for PSM and 0.87 for MSM. In the present study, Cronbach’s alphas were 0.88 for PSM and 0.82 for MSM.

#### 2.2.3 Impact of event scale-revised (IES-R)

IES-R (Lopes and Rocha, 2013) measures PTSS, i.e., symptoms associated with exposure to traumatic events. This scale comprises 22 items across three subscales: intrusion, avoidance, and hyperarousal. Participants were asked to rate each item based on their experiences during the past week, using a five-point scale. The global score was used in this study, with higher scores reflecting a higher number of PTSS. Cronbach’s alpha for the Portuguese IES-R version was 0.95, while in this study it was 0.97.

#### 2.2.4 Event-related rumination inventory (ERRI)

This scale comprises 20 items that measure two rumination styles: intrusive thoughts and deliberate rumination (Ramos et al., 2015). Participants were instructed to rate each item based on their thoughts during the weeks immediately after hospital discharge, using a four-point Likert scale. The present study used the total score, with higher results indicating a higher frequency of intrusive and deliberate ruminations. The internal consistency of the Portuguese version was 0.94, and in this study, it was 0.97.

#### 2.2.5 Hospital anxiety and depression scale (HADS)

HADS (Pais-Ribeiro et al., 2007) consists of 14 items that assess psychological morbidity, specifically symptoms of depression and anxiety. Each item in the scale is assigned a score of 0–3, with higher scores indicating greater symptom severity or psychological morbidity. Cronbach’s alphas for the Portuguese version were 0.81 for depression and 0.76 for anxiety. The present study used the total score, yielding a Cronbach’s alpha of 0.87.

#### 2.2.6 UCLA loneliness Scale-16 (UCLA)

UCLA (Faustino et al., 2019) consists of 16 items used to assess subjective feelings of loneliness, divided into two factors: social isolation and affinity. Items are rated on a four-point Likert scale, with higher scores indicating a greater perceived level of loneliness. This study used the global scale, which has a Cronbach’s alpha of 0.93 for both the Portuguese version and this study.

#### 2.2.7 Satisfaction with life scale (SWLS)

SWLS (Reppold et al., 2019) is a self-report instrument composed of five items that assess an individual’s global cognitive judgments of life satisfaction. Participants indicate their level of agreement with each item using a five-point Likert scale. Higher scores reflect greater overall life satisfaction. Cronbach’s alpha for the Portuguese version was 0.77, and in the current sample, it was 0.82.

#### 2.2.8 Posttraumatic growth inventory (PGI)

PGI (Lamela et al., 2013) comprises 10 items to assess personal growth following adversity. Items are divided into five dimensions: relating to others, new possibilities, personal strength, spiritual change, and appreciation of life. Participants rated the extent to which they experienced each change during hospitalization using a six-point Likert scale. This study used the global score, with higher scores reflecting greater PTG. Internal consistency for the total score in the Portuguese version was 0.88, and in this study was 0.94.

#### 2.2.9 Physiological markers

The patient’s blood samples were collected through a peripheral catheter. Blood cell counts were performed using the Sysmex XN-9000™ (Sysmex Corporation). For biochemical determinations to assess CRP levels, a latex-enhanced immunoturbidimetric method was used. Plasma fibrinogen levels were measured with the Clauss method using an ACL TOP 500 analyzer (Werfen, Barcelona, Spain). All analyses were conducted according to the manufacturer’s protocols and standard laboratory procedures.

#### 2.2.10 Real-time PCR analysis for the expression of DR in PBMCs

PBMCs were isolated using density gradient centrifugation (Ficoll method), and DR was assayed by real-time PCR as previously described (Zaffaroni et al., 2008). Briefly, total RNA was extracted from PBMCs using the PerfectPure RNA Cell & Tissue kit (5Prime), and RNA yield was determined by spectrophotometry at 260 nm. Total RNA was then reverse transcribed using the High-Capacity cDNA Archive Kit (Applied Biosystems, Foster City, USA), according to the manufacturer’s instructions. Real-time PCR was performed using an ABI Prism 7,000 apparatus (Applied Biosystems) with Assay on Demand kits for the genes of interest (Applied Biosystems), following the manufacturer’s instructions. Gene sequence data were obtained from the Reference Sequence collection (RefSeq; www.ncbi.nlm.nih.gov/projects/RefSeq). Relative expression was determined by normalization to 18S rRNA (housekeeping gene) using AB Prism 7,000 SDS software. Detailed information on RNA quality control, technical replicates, Ct thresholds, melting curve analysis, and the relative quantification method is provided in Supplementary material S1.

### 2.3 Data analysis

Demographic and clinical data were analyzed using frequencies (percentages) for categorical variables and means (standard deviations) for continuous variables. Independent sample t-tests were conducted to assess differences in QoL between patients hospitalized with COVID-19 as the primary *versus* secondary diagnosis. Pearson correlation coefficients were used to analyze the relationships among variables.

For the path analysis, only mediator variables (psychosocial, inflammatory, and dopaminergic markers) that showed significant correlations with either physical or mental QoL (*p* < 0.05) were included (Hair et al., 2010; Kline, 2015). DRD4 was also included as a mediator, as it was significantly correlated with an independent variable (PTSS) and two mediator variables (DRD1 and NLR), as shown in Table 1 (Hayes, 2018). The final model retained only DRD1 and DRD4 as mediators. Both theoretical and empirical considerations guided this selection, as these receptors are key regulators of stress reactivity, prefrontal dopaminergic signaling, and immune modulation (Cosentino and Marino, 2013; Lauzon et al., 2012, 2013; Wang et al., 2018; Yan et al., 2015), and they showed significant associations with psychosocial and inflammatory variables in preliminary correlation analyses (Table 1). A power analysis was conducted with power set at 0.80, a significance level of 5%, a medium effect size, and nine independent variables (PTSS, rumination, psychological morbidity, loneliness, satisfaction with life, PTG, DRD1, DRD4, and NLR), requiring a sample size of 113 participants (Soper, 2019).

**Table 1:** Correlations between variables.

	1. Physical QoL	2. Mental QoL	3. PTSS	4. Rumination	5. Psych. morbidity	6. Loneliness	7. Satisfaction with life	8. PTG	9. NLR	10. Ferritin	11. CRP	12. DRD1	13. DRD3	14. DRD4	15. DRD5
1. Physical QoL	1														
2. Mental QoL	0.696***	1													
3. PTSS	−0.190*	−0.348***	1												
4. Rumination	−0.221**	−0.380***	0.784***	1											
5. Psych. morbidity	−0.552***	−0.747***	0.431***	0.505***	1										
6. Loneliness	−0.211**	−0.424***	0.159*	0.241***	0.509***	1									
7. Satisfaction with life	0.338***	0.433***	−0.197*	−0.238***	−0.481***	−0.331***	1								
8. PTG	0.010	−0.160*	0.398***	0.546***	0.272***	0.188*	0.063	1							
9. NLR	−0.165*	−0.011	−0.103	−0.100	−0.019	0.029	−0.083	−0.073	1						
10. Ferritin	0.074	0.043	−0.042	−0.106	−0.153*	−0.056	−0.095	−0.124	0.152*	1					
11. CRP	−0.121	−0.107	−0.061	−0.082	0.050	−0.074	−0.091	−0.015	0.029	−0.043	1				
12. DRD1	0.170*	0.092	0.154*	0.081	−0.108	−0.096	0.098	−0.007	−0.157*	−0.003	0.060	1			
13. DRD3	−0.083	0.007	0.060	0.001	0.030	0.011	−0.038	0.080	0.160*	0.144*	0.003	−0.098	1		
14. DRD4	0.099	0.038	0.218**	0.132	−0.026	−0.014	0.038	0.047	−0.154*	−0.007	−0.022	0.636***	−0.123	1	
15. DRD5	−0.021	0.132	−0.100	−0.038	−0.017	−0.130	0.069	0.066	0.113	−0.041	−0.033	−0.412***	0.354***	−0.349***	1

QoL, quality of life; PTSS, posttraumatic stress symptoms; PTG, posttraumatic growth; NLR, neutrophil-to-lymphocyte ratio; CRP, C-reactive protein; *p < 0.05; **p < 0.01; ***p < 0.001.

Model fit was assessed using the chi-square statistic (*χ*^2^), Goodness of Fit Index (GFI), Comparative Fit Index (CFI), Root Mean Square Error of Approximation (RMSEA), and Standardized Root Mean Square Residual (SRMR). A *χ*^2^/df ratio below 2, GFI and CFI values equal to or greater than 0.95, RMSEA below 0.07, and SRMR below 0.08 indicated a good fit (Hair et al., 2010). To mitigate the risk of Type I errors associated with the partially exploratory nature of the model, bootstrapping with 5,000 samples and 95% bias-corrected confidence intervals was applied to all parameter estimates (direct and indirect paths). Additionally, multigroup analyses were conducted to test the moderating effect of time since discharge (cohort I *versus* cohort II). Statistical significance was set at a *p*-value of < 0.05. Statistical analyses were conducted using SPSS (version 29.0) and AMOS (version 29.0).

## 3 Results

### 3.1 Sample characteristics

This study included 207 participants (130 male), with an average age of 61.65 years (*SD* = 10.59). All patients had been hospitalized with COVID-19 as the primary diagnosis (*n* = 145; 70%) or secondary diagnosis (*n* = 62; 30%). There were no statistically significant differences between these groups in physical [*t* (205) = 0.134, *p* = 0.894] and mental [*t* (205) = 0.963, *p* = 0.337] QoL. Sociodemographic and clinical characteristics of the sample are presented in Table 2.

**Table 2:** Participants’ sociodemographic and clinical characteristics (*n* = 207).

Participants’ characteristics	Total (*N* = 207)		Cohort I (*n* = 78)		Cohort II (*n* = 129)	
	*n* (%)	Mean (*SD*)	*n* (%)	Mean (*SD*)	*n* (%)	Mean (*SD*)
Gender						
Men	130 (62.8)		49 (62.8)		81 (62.8)	
Women	77 (37.2)		29 (37.2)		48 (37.2)	
Age		61.65 (10.59)		63.82 (9.27)		60.34 (11.14)
BMI (kg/m^2^)		28.26 (5.17)		29.48 (4.81)		27.54 (5.25)
With partner						
No	79 (38.2)		28 (35.9)		51 (39.5)	
Yes	128 (61.8)		50 (64.1)		78 (60.5)	
Education						
With higher education	25 (12.1)		13 (16.7)		12 (9.3)	
Without higher education	182 (87.9)		65 (83.3)		117 (90.7)	
Employment status						
Inactive	137 (66.2)		47 (60.3)		90 (69.8)	
Active	70 (33.8)		31 (39.7)		39 (30.2)	
Pre-existing medical condition(s)						
No	13 (6.3)		7 (9.0)		6 (4.7)	
Yes	194 (93.7)		71 (91.0)		123 (95.3)	
Primary hospitalization diagnosis						
COVID-19	145 (70.0)		77 (98.7)		68 (52.7)	
Other	62 (30.0)		1 (1.3)		61 (47.3)	
Admission to the ICU						
No	163 (78.7)		61 (78.2)		102 (79.1)	
Yes	44 (21.3)		17 (21.8)		27 (20.9)	
Length of ICU stay (in days)		10.02 (10.46)		10.71 (7.82)		9.58 (12.01)
Hospitalization cohort						
Cohort I	78 (37.7)					
Cohort II	129 (62.3)					
Length of hospital stay (in days)		13.85 (18.42)		12.72 (11.30)		14.53 (21.63)
COVID severity		4.02 (0.90)		4.32 (0.67)		3.84 (0.98)

BMI, body mass index; ICU, intensive care unit.

### 3.2 Path analysis model

**Figure 3:**
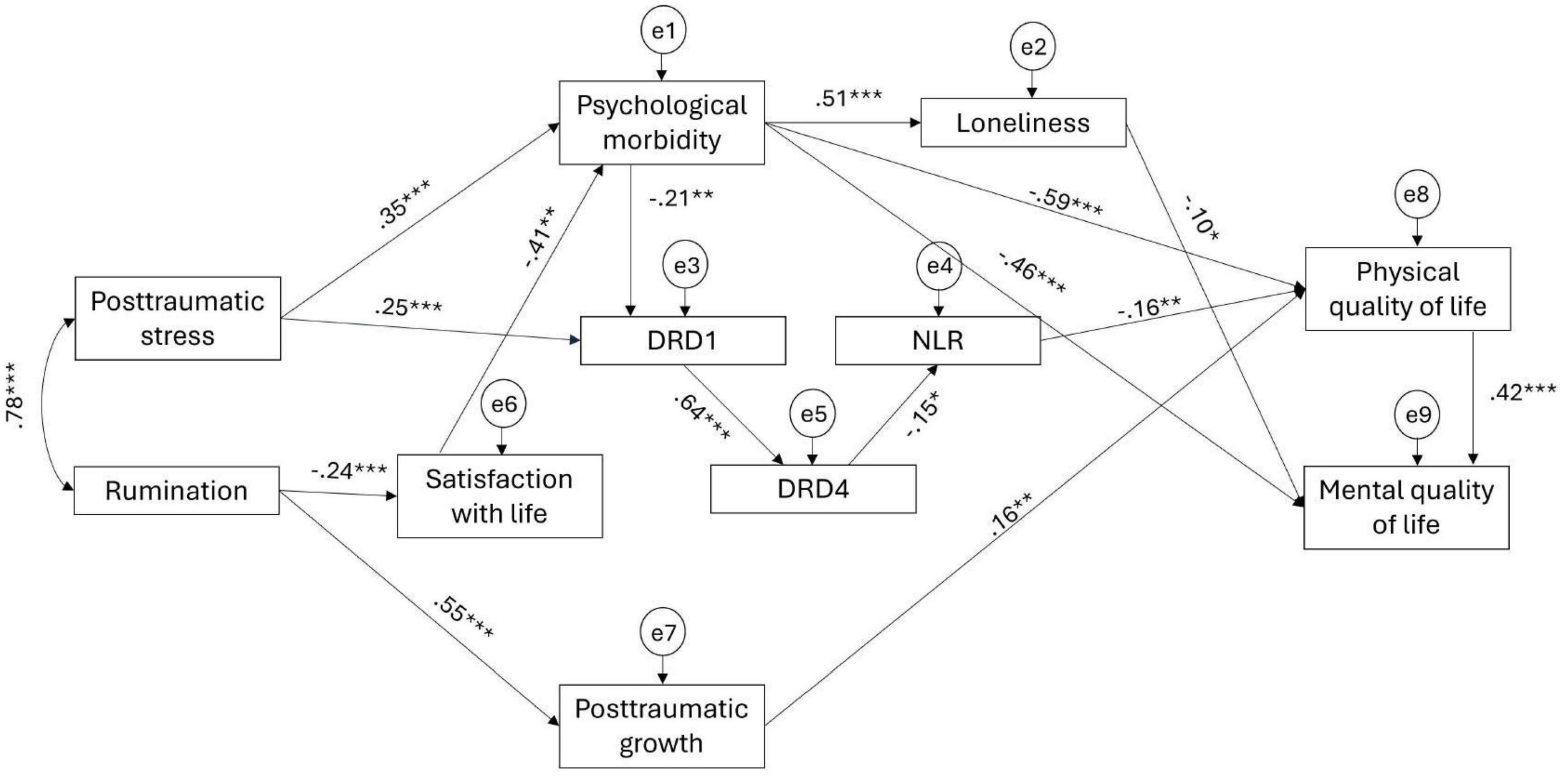
Final adjusted model.

The initial proposed model showed no adjustment to the data, as the values of the fit indices were not adequate: *χ*^2^/DF = 7.86, GFI = 0.80, CFI = 0.70, RMSEA = 0.18 [0.16, 0.20], and SRMR = 0.14. Subsequently, several pathways were explored according to the modification indices, the significance of the path coefficients, and the final model adjustment. Therefore, after removing non-significant paths (*p* < 0.05), the remaining modification indices were analyzed and included, as they were theoretically supported and improved the overall model fit. These modifications resulted in the addition of direct relationships among mediators (psychological morbidity → loneliness; DRD1 → DRD4; satisfaction with life → psychological morbidity; PTG → physical QoL) and dependent variables (physical QoL → mental QoL) (Figure 3). The final adjusted model provided a good fit to the data: *χ*^2^/DF = 1.237, GFI = 0.960, CFI = 0.989, RMSEA = 0.034 [0.000, 0.062], and SRMR = 0.051 (Figure 3).

All pathways in the adjusted model were statistically significant except for the path between DRD4 and RNL (Table 3). Although the parametric estimate indicated a significant negative association between these variables (*β* = −0.154, *p* = 0.025), the bias-corrected bootstrap confidence interval crossed zero, so this pathway cannot be considered statistically reliable. PTSS was positively associated with psychological morbidity and DRD1 expression. The association between rumination and PTG was more than twice as stronger as that observed between rumination and satisfaction with life. Physical QoL was negatively associated with psychological morbidity and NLR, and positively associated with PTG. Mental QoL was positively associated with physical QoL, and negatively associated with psychological morbidity and loneliness.

**Table 3:** Standardized direct paths in the final adjusted model.

Path	*β*	CI_95%_
PTSS → psychological morbidity	0.350***	[0.201, 0.498]
PTSS → DRD1	0.247***	[0.119, 0.376]
Rumination → satisfaction with life	−0.238***	[−0.363, −0.115]
Rumination → PTG	0.546***	[0.417, 0.653]
Psychological morbidity → DRD1	−0.214**	[−0.367, −0.058]
Psychological morbidity → loneliness	0.509***	[0.396, 0.610]
Psychological morbidity → physical QoL	−0.594***	[−0.685, −0.486]
Psychological morbidity → mental QoL	−0.461***	[−0.575, −0.347]
Satisfaction with life → psychological morbidity	−0.412***	[−0.522, −0.290]
PTG → physical QoL	0.160*	[0.051, 0.267]
DRD1 → DRD4	0.636***	[0.534, 0.715]
DRD4 → NLR	−0.154*	[−0.301, 0.042]
Loneliness → mental QoL	−0.099*	[−0.197, −0.002]
NLR → physical QoL	−0.164**	[−0.260–0.042]
Physical QoL → mental QoL	0.419***	[0.320, 0.509]

*p < 0.05; **p < 0.005; ***p < 0.001; *β* = standardized path coefficient; CI_95_% = Bootstrap bias-corrected confidence interval at 95% (5,000 samples), lower and upper.

The indirect paths between PTSS and both physical and mental QoL were statistically significant. However, the intermediary indirect path between PTSS and NLR did not reach statistical significance. Regarding the indirect paths between rumination and QoL, neither effect was significant. Nevertheless, all intermediary indirect paths showed statistical significance, except for the path between rumination and NLR (Table 4).

**Table 4:** Standardized indirect paths in the final adjusted model.

Indirect path			*β*	CI_95%_
PTSS	Psych. morbidity	Physical QoL	−0.205***	[−0.316, −0.110]
	Psych. morbidity → DRD1 → DRD4 → NLR			
	DRD1 → DRD4 → NLR			
PTSS	Psych. morbidity	Mental QoL	−0.265***	[−0.390, −0.146]
	Psych. morbidity → loneliness			
	DRD1 → DRD4 → NLR → physical QoL			
	Psych. morbidity → DRD1 → DRD4 → NLR → Physical QoL			
PTSS	Psych. morbidity	Loneliness	0.178***	[0.102, 0.267]
PTSS	Psych. morbidity	DRD1	−0.075*	[−0.154, −0.023]
PTSS	Psych. morbidity → DRD1	DRD4	0.109**	[0.040, 0.180]
	DRD1			
PTSS	Psych. morbidity → DRD1 → DRD4	NLR	−0.017	[−0.046, 0.002]
	DRD1 → DRD4			
Rumination	Satisfaction with life → Psych. morbidity	Physical QoL	0.029	[−0.042, 0.108]
	Satisfaction with life → Psych. morbidity → DRD1 → DRD4 → NLR			
	PTG			
Rumination	Satisfaction with life → Psych. morbidity	Mental QoL	−0.038	[−0.096, 0.015]
	Satisfaction with life → Psych. morbidity → physical QoL			
	Satisfaction with life → Psych. morbidity → loneliness			
	Satisfaction with life → Psych. morbidity → DRD1 → DRD4 → NLR → physical QoL			
	PTG → physical QoL			
Rumination	Satisfaction with life	Psych. morbidity	0.098**	[0.043, 0.169]
Rumination	Satisfaction with life	Loneliness	0.050**	[0.021, 0.093]
Rumination	Satisfaction with life	DRD1	−0.021*	[−0.048, −0.006]
Rumination	Satisfaction with life → DRD1	DRD4	−0.013*	[−0.032, −0.003]
Rumination	Satisfaction with life → DRD1 → DRD4	NLR	0.002	[0.000, 0.008]

*p < 0.05; **p < 0.005; ***p < 0.001; *β* = standardized regression coefficient; CI_95_% = Bootstrap bias-corrected confidence interval at 95% (5,000 samples), lower and upper.

### 3.3 The moderating role of time since discharge

The analysis of the moderating effect of time since discharge, comparing patients from cohort I (discharged 24 months ago) *versus* cohort II (discharged 6–12 months ago), showed significant differences between the adjusted model and the fully constrained model [*Δχ*^2^ (15) = 36.425; *p* = 0.002], indicating that cohorts significantly moderated the hypothesized relationships in the model. In particular, patients from cohort I showed a significant relationship between PTG and physical QoL (*β* = 0.216, *p* = 0.023). Conversely, the associations between PTSS and DRD1 (*β* = 0.268, *p* = 0.006), DRD1 and DRD4 (*β* = 0.691, *p* < 0.001), NLR and physical QoL (*β* = −0.191, *p* = 0.006), loneliness and mental QoL (*β* = −0.199, *p* < 0.001), and rumination and satisfaction with life (*β* = −0.293, *p* < 0.001) were significant only for the most recently discharged patients (cohort II).

Analysis of the indirect paths revealed significant associations in both cohorts, although cohort II exhibited a greater number of significant indirect paths overall. The indirect path linking PTG with mental QoL was statistically significant only in cohort I (*β* = 0.114, *p* = 0.028), suggesting that this association may be more relevant for patients with a longer time since discharge. In contrast, multiple indirect paths were uniquely significant in cohort II, such as the indirect association between rumination and psychological morbidity (*β* = 0.119, *p* = 0.001), DRD1 (*β* = −0.031, *p* = 0.011), DRD4 (*β* = −0.021, *p* = 0.010), and loneliness (*β* = 0.026, *p* < 0.001). Moreover, only patients from cohort II showed significant indirect paths between PTSS and DRD1 (*β* = −0.100, *p* = 0.013)/DRD4 (*β* = 0.116, *p* = 0.033); satisfaction with life and DRD1 (*β* = 0.104, *p* = 0.017)/DRD4 (*β* = 0.072, *p* = 0.017); psychological morbidity and DRD4 (*β* = −0.177, *p* = 0.019); and NLR and mental QoL (*β* = −0.070, *p* = 0.014). Finally, among the indirect paths that were significant in both cohorts, cohort II consistently showed larger coefficients, underscoring more pronounced psychological, inflammatory, and dopaminergic dynamics among patients discharged more recently.

## 4 Discussion

To the best of our knowledge, this is the first study to integrate psychosocial, inflammatory, and dopaminergic factors into a unified path analysis model examining post-hospitalization health-related outcomes in patients with COVID-19. This work proposes a novel, multidimensional framework for understanding the role of psychological morbidity (anxiety and depressive symptoms), dopaminergic signaling (DRD1 and DRD4 expression), and inflammation (NLR) in the relationship between maladaptive psychological responses (PTSS and rumination) and QoL. Moreover, the analysis of the moderating role of time since hospital discharge offers novel insights into the potential temporal dynamics of recovery, further highlighting the interaction between psychosocial and neurobiological processes.

Findings regarding psychosocial factors align with previous research on hospitalized COVID-19 patients and long-COVID symptoms. For instance, PTSS was significantly associated with anxiety and depressive symptoms, highlighting its enduring role as a risk factor for poor mental health post-discharge (Engelmann et al., 2024; Evans et al., 2021; Vlake et al., 2021). As expected, rumination emerged as a relevant factor, showing a strong positive association with PTG, consistent with the finding that, following severe illness, some patients typically experience positive psychological changes (Özgüç et al., 2022). Furthermore, satisfaction with life played a key role in the relationship between rumination and psychological morbidity, whereby higher rumination was associated with reduced satisfaction with life, which, in turn, was related to higher levels of anxiety and depression. This indirect association, although small, suggests that satisfaction with life may represent a potential protective factor against distress. In this line, psychological morbidity was linked to poorer QoL and greater loneliness, consistent with recent evidence linking social isolation and mental health burden in discharged patients with prolonged COVID-19 symptoms (Mazza et al., 2020; Müller et al., 2025). Physical QoL was positively related to mental QoL, while loneliness was negatively associated with mental QoL, underscoring the interconnectedness of physical health, emotional resilience, and social support (Huang et al., 2023). It should be noted that, although participants displayed psychological symptoms commonly associated with long COVID-19 (National Institute for Health and Care Excellence, 2020), the study did not assess whether they met the clinical criteria for post-COVID-19 condition.

DR expression in PBMCs, particularly DRD1 and DRD4, was significantly associated with psychological and physiological outcomes. The focus on DRD1 and DRD4 was theoretically grounded rather than exclusively data-driven. These receptors represent the two dopaminergic subtypes most consistently linked to stress-related neurocircuitry and immune regulation. DRD1 activation has been shown to inhibit the NLRP3 (NOD-like receptor protein 3) inflammasome and reduce pro-inflammatory signaling (Yan et al., 2015; Wang et al., 2018), while DRD4 modulates CaMKII-dependent synaptic plasticity and emotional memory processing within the prefrontal cortex (Lauzon et al., 2012). Moreover, both receptors have been associated with dopaminergic–immune cross-talk in peripheral immune cells (Cosentino and Marino, 2013). The expression of DRD1 and DRD4 showed coordinated regulation, while lower DRD1 expression in PBMCs was linked to greater psychological morbidity, highlighting dopaminergic dysregulation as a potential marker of vulnerability in potentially trauma-related conditions. PTSS has been consistently linked to dysregulation in fear-related neural circuits, particularly involving the prefrontal cortex (PFC), amygdala, and hippocampus (Bainter et al., 2024). The DA system, especially dopaminergic neurons in the ventral tegmental area (VTA), is structurally and functionally connected to this posttraumatic stress-related synaptic circuit (Liu J. et al., 2023). Although direct evidence in PTSS is limited, dysregulated DA transmission from the VTA to the medial PFC and hippocampus has been linked to posttraumatic stress disorder (PTSD; Torrisi et al., 2019; Zhou et al., 2021). Moreover, genetic alterations in DR and the DA reuptake protein, the DA transporter, may lead to PTSS (Zuschlag et al., 2021) and neuroinflammation (Illiano et al., 2021).

To the best of our knowledge, this is the first study to report a relationship between DRD1 and DRD4 expression in PBMCs and PTSS in humans. In fact, DA, in addition to its central role as a classical neurotransmitter, modulates peripheral immune function (Sarkar et al., 2010; Cosentino and Marino, 2013). Multiple studies support the use of PBMCs as a valuable source of biomarkers for human disease (Rahmoune and Guest, 2018; Pansarasa et al., 2022), particularly in psychiatric diseases (Pansarasa et al., 2022; Saponjic et al., 2024). Azadmarzabadi et al. (2018) reported upregulation of DRD1 and DRD4 in peripheral blood samples from an Iranian cohort, linking these changes to reduced stress resilience and distinct psychological profiles, including elevated psychological morbidity. In the present study, DRD1 expression was positively associated with PTSS but inversely related to psychological morbidity, suggesting a complex, possibly bidirectional role for DRD1 in stress-related outcomes. While preclinical models, such as the single-prolonged stress paradigm, have not consistently shown alterations in DRD1 in the striatum (Enman et al., 2015), evidence from PTSD research suggests that DRD1 modulates memory circuits through cAMP signaling in the PFC (Carrion et al., 2009), providing a mechanistic basis for similar associations in PTSS.

The small negative association between DRD4 and NLR was not statistically significant, contrary to expectations, despite NLR being an important prognostic marker in COVID-19 patients (Ulloque-Badaracco et al., 2021). While previous studies suggest that DA can supress NLRP3, leucine-rich repeat (LRR), and pyrin domain-containing protein 3 (NLRP3) inflammasome activation via DRD1 pathways (Yan et al., 2015; Wang et al., 2018), our findings indicate that DRD4 may not mediate this mechanism in the context of post-COVID-19 recovery. Under chronic stress or PTSS, reduced DA availability may favor activation of high-affinity receptors, potentially enhancing neuroinflammatory responses (Gaskill and Khoshbouei, 2022), which could partly explain the lack of a significant DRD4–NLR association in our sample. Given that PBMCs can reflect neuroimmune dynamics (Rahmoune and Guest, 2018), DRD1 and DRD4 may represent promising peripheral markers of stress-related pathophysiology. However, given the exploratory nature of our model, further investigation is warranted to clarify the specific contribution of dopaminergic pathways to the regulation of inflammatory responses in this population.

Time since discharge, across the two cohorts, moderated the adjusted model, thereby highlighting the key role of time after hospitalization in the psychological and physiological pathways underlying mental and physical QoL. The relationship between PTG and physical QoL was significant only among patients discharged for a longer period (24 months), suggesting that PTG may play a more prominent role in patients’ long-term physical recovery. Previous research during the COVID-19 pandemic found that higher levels of PTG significantly predicted better physical QoL but not mental QoL (Fillion, 2024). Furthermore, the association between PTG and physical QoL over the 24 months may reflect the gradual integration of growth-related changes into daily behavior and self-care routines, which may not immediately relate to psychological well-being. The absence of a significant relationship with mental QoL in cohort I may suggest that mental health outcomes may require additional time and different psychosocial mechanisms for improvement.

Patients from cohort II showed significant negative associations between loneliness and mental QoL and between rumination and satisfaction with life, suggesting that these associations may weaken over time. In fact, the early post-discharge phase may represent a particularly vulnerable period for COVID-19 patients, marked by an increased risk of adverse psychological outcomes and reduced overall QoL (Huang et al., 2021). During this period, psychosocial recovery may also be influenced by external factors such as COVID-19 stigma, social isolation, and access inequities in digital mental health resources. Stigmatization may intensify feelings of loneliness and discourage help-seeking behaviors, while limited access to telemedicine or online support may hinder timely interventions after discharge (Datta et al., 2022; Duan et al., 2020). Further longitudinal studies are warranted to investigate changes in patients’ recovery trajectories post-discharge, with particular attention to how social and ethical determinants interact with psychosocial and cognitive–emotional factors to elucidate the causal relationships between these constructs.

Elevated systemic inflammation, as measured via NLR levels, was associated with lower physical QoL only in cohort II patients, suggesting that the link between inflammatory processes and physical health may decrease over time or interact with other recovery-related factors (Furman et al., 2019). Although small, this significant association highlights the potential value of NLR as a short-term prognostic marker of physical well-being and supports the role of inflammation as a possible therapeutic target in post-COVID recovery.

Patients from cohort II showed several significant indirect pathways, whereas in cohort I, only the indirect path between PTG and mental QoL was statistically significant. In fact, PTG is a time-dependent process that requires reflective capacity to facilitate growth, adaptation, and psychological change following exposure to a potentially traumatic event (Lamela et al., 2013). Shortly after hospital discharge, COVID-19 survivors often deal with more immediate psychological and physical sequelae of their illness, which may hinder the process of PTG. For instance, a systematic review and meta-analysis of one-year follow-up studies on post-COVID-19 symptoms showed that patients continued to experience significant physical (e.g., fatigue, arthromyalgia), psychological (e.g., depression, anxiety), and cognitive (e.g., memory loss) impairments within 12 months after discharge (Han et al., 2022).

Regarding cohort II, the significant indirect paths between rumination and psychosocial functioning (psychological morbidity and loneliness) were expected. In this study, rumination was assessed using the ERRI, which measures both intrusive thoughts and deliberate rumination associated with PTG (Ramos et al., 2015). As previously mentioned, these processes are time-dependent. Previous studies have also found that rumination decreases over time and is associated with loneliness, anxiety, and depressive symptoms, especially among patients with pre-existing mental health conditions (O’Connor et al., 2022).

The indirect paths between rumination and DRD1/DRD4 expression in PBMCs were significant only in cohort II, suggesting that cognitive processes, such as repetitive negative thinking, may relate to dopaminergic pathways in recently discharged COVID-19 patients. Additionally, the associations between PTSS and DRD1/DRD4 expression, as well as between psychological morbidity and DRD4, are consistent with dopaminergic involvement in psychopathology (Cheng et al., 2020). Moreover, the significant indirect path between satisfaction with life and DRD1/DRD4 expression in this cohort highlights a potential association between dopaminergic signaling and positive psychological outcomes shortly after discharge. Finally, the indirect path between NLR and mental QoL suggests that systemic inflammation may be related to mental well-being via intermediary pathways in the early post-discharge period. Future studies should consider the roles of key cytokines (e.g., interleukin-6 and interleukin-10) to better elucidate the mechanistic pathways linking dopaminergic signaling and inflammatory processes. While statistically significant, the indirect effects observed in both cohorts were modest, suggesting subtle associations among the variables.

### 4.1 Limitations

This study presents some limitations that should be acknowledged. First, the relatively small sample size and the fact that data were collected in a single hospital (although a leading academic central hospital) may limit the generalization of the findings. In addition, only patients who provided informed consent and complete data were included in the study. Given that no information was available for non-participants, potential selection bias cannot be ruled out, which may further limit the external validity of the results. Second, the heterogeneity of the sample regarding sociodemographic (e.g., age, gender) and clinical (e.g., hospitalization duration, ICU admission, BMI) characteristics may have influenced the outcomes. For example, 30% of participants were hospitalized with COVID-19 as a secondary diagnosis, which, despite the absence of statistically significant differences in the outcome variables, introduces additional clinical heterogeneity that may have affected the results. Third, the omission of potential confounding sociodemographic (e.g., socioeconomic status) and clinical factors (e.g., medication use, vaccination status, reinfection history) further limits the robustness of the conclusions. In particular, given that 94% of participants had pre-existing medical conditions, a more detailed analysis of comorbidities would be warranted, as these factors may have influenced the findings. Additionally, it is worth noting that the two cohorts correspond to different phases of the pandemic; therefore, contextual factors such as circulating variants, treatment protocols, and societal stress levels may have contributed to the observed differences.

Finally, the cross-sectional design does not allow for causal inferences, and the exploratory nature of the model limits the strength of the conclusions. As a result, the findings should be interpreted cautiously and validated through replication in subsequent studies. Future longitudinal studies with larger and more balanced samples are needed to clarify potential causal relationships, and enhance the robustness of the findings.

### 4.2 Implications for clinical practice

The present findings show the importance of integrated biopsychosocial care as part of COVID-19 treatment and recovery. Discharged patients, besides systemic inflammation, may experience multifaceted needs, including significant psychological morbidity, traumatic reactions, and alterations in dopaminergic pathways. The interaction of these factors may contribute to poorer mental and physical QoL, and hinder overall recovery. Early psychosocial screening targeting psychological distress, loneliness, and maladaptive cognitive patterns (e.g., rumination) may help identify patients who may benefit from targeted interventions, thereby mitigating downstream effects on inflammatory and dopaminergic systems, and influencing both mental health and biological recovery markers.

Moreover, interventions that enhance dopaminergic signaling hold promise as therapeutic agents. For instance, L-DOPA has been shown to improve spontaneous reactivation within the ventromedial prefrontal cortex and amygdala, thereby strengthening dopaminergic pathways. A completed clinical trial demonstrated that L-DOPA administered after extinction learning increased amygdala reactivation and reduced fear reinstatement in women with posttraumatic stress disorder, suggesting its potential to improve the consolidation of extinction memories and reduce the relapse of fear responses (Cisler et al., 2020). While further research is needed, our results point to similar dopaminergic interventions addressing trauma-related psychological symptoms in patients recovering from COVID-19. Additionally, non-pharmacological strategies, including lifestyle-based approaches, may support dopaminergic function and neurobiological resilience. Integrating these approaches with neurobiological monitoring could improve QoL and reduce the risk of chronic physical and psychological sequelae among post-COVID-19 patients.

## 5 Conclusion

The results of the present study reveal a complex interplay among psychosocial, inflammatory, and dopaminergic factors affecting QoL after COVID-19 hospitalization, suggesting that the acute phase following hospital discharge represents a critical window for intervention. The findings underscore the need for timely, multidimensional interventions tailored to various stages of recovery, encompassing both psychosocial and neurobiological mechanisms to foster long-term mental and physical well-being. Healthcare professionals should be aware of the interconnected psychological, social, and neurobiological factors affecting patients after COVID-19, including stress-related symptoms and dopaminergic signaling. An integrated care approach combining psychological interventions and support with attention to biological markers may help healthcare providers optimize long-term recovery and QoL in post-COVID patients.

Overall, the results emphasize the importance of longitudinal designs in clarifying evolving recovery patterns, and supporting the development of tailored interventions that consider both the timing and nature of post-COVID-19 adjustment processes.

## Data Availability

The data are available and can be provided upon request to the corresponding author.

## References

[ref1] Alzahrani N. (2021). The effect of hospitalization on patients' emotional and psychological well-being among adult patients: an integrative review. Appl. Nurs. Res. 61:151488. doi: 10.1016/j.apnr.2021.151488, 34544571

[ref2] Azadmarzabadi E. Haghighatfard A. Mohammadi A. (2018). Low resilience to stress is associated with candidate gene expression alterations in the dopaminergic signaling pathway. Psychogeriatrics 18, 190–201. doi: 10.1111/psyg.12312 29423959

[ref3] Bainter S. A. Goodman Z. T. Kupis L. B. Timpano K. R. Uddin L. Q. (2024). Neural and psychological correlates of post-traumatic stress symptoms in a community adult sample. Cereb. Cortex 34:bhae214. doi: 10.1093/cercor/bhae214, 38813966 PMC12536900

[ref4] Carrion V. G. Weems C. F. Watson C. Eliez S. Menon V. Reiss A. L. (2009). Converging evidence for abnormalities of the prefrontal cortex and evaluation of midsagittal structures in pediatric posttraumatic stress disorder: an MRI study. Psychiatry Res. Neuroimaging 172, 226–234. doi: 10.1016/j.pscychresns.2008.07.008, 19349151 PMC2704559

[ref5] Ceban F. Ling S. Lui L. M. W. Lee Y. Gill H. Teopiz K. M. . (2022). Fatigue and cognitive impairment in post-COVID-19 syndrome: a systematic review and meta-analysis. Brain Behav. Immun. 101, 93–135. doi: 10.1016/j.bbi.2021.12.020, 34973396 PMC8715665

[ref6] Cheng P. W. C. Chang W. C. Lo G. G. Chan K. W. S. Lee H. M. E. Hui L. M. C. . (2020). The role of dopamine dysregulation and evidence for the transdiagnostic nature of elevated dopamine synthesis in psychosis: a positron emission tomography (PET) study comparing schizophrenia, delusional disorder, and other psychotic disorders. Neuropsychopharmacology 45, 1870–1876. doi: 10.1038/s41386-020-0740-x, 32612207 PMC7608388

[ref7] Cisler J. M. Privratsky A. A. Sartin-Tarm A. Sellnow K. Ross M. Weaver S. . (2020). L-DOPA and consolidation of fear extinction learning among women with posttraumatic stress disorder. Transl. Psychiatry 10:287. doi: 10.1038/s41398-020-00975-3, 32801342 PMC7429959

[ref8] Cosentino M. Marino F. (2013). Adrenergic and dopaminergic modulation of immunity in multiple sclerosis: teaching old drugs new tricks? J. Neuroimmune Pharmacol. 8, 163–179. doi: 10.1007/s11481-012-9410-z, 23074017

[ref9] Datta P. Eiland L. Samson K. Donovan A. Anzalone A. J. McAdam-Marx C. (2022). Telemedicine and health access inequalities during the COVID-19 pandemic. J. Glob. Health 12:05051. doi: 10.7189/jogh.12.05051, 36462207 PMC9718446

[ref10] Di Tella M. Romeo A. (2025). Posttraumatic stress symptoms and rumination: the moderator effect of time. Psychol. Health Med. 30, 697–707. doi: 10.1080/13548506.2024.2433542, 39612938

[ref11] Duan W. Bu H. Chen Z. (2020). COVID-19-related stigma profiles and risk factors among people who are at high risk of contagion. Soc. Sci. Med. 266:113425. doi: 10.1016/j.socscimed.2020.113425, 33059301 PMC7540249

[ref12] Eidhof I. Twohig D. Falk A. (2024). Unraveling the brain’s response to COVID-19: how SARS-CoV-2 afflicts dopaminergic neurons. Cell Stem Cell 31, 152–154. doi: 10.1016/j.stem.2024.01.002. 38306990

[ref13] Engelmann P. Reinke M. Stein C. Salzmann S. Löwe B. Toussaint A. . (2024). Psychological factors associated with long COVID: a systematic review and meta-analysis. EClinicalMedicine 74:102756. doi: 10.1016/j.eclinm.2024.102756, 39764180 PMC11701445

[ref14] Enman N. M. Arthur K. Ward S. J. Perrine S. A. Unterwald E. M. (2015). Anhedonia, reduced cocaine reward, and dopamine dysfunction in a rat model of posttraumatic stress disorder. Biol. Psychiatry 78, 871–879. doi: 10.1016/j.biopsych.2015.04.024, 26115790 PMC4644715

[ref15] Evans R. A. McAuley H. Harrison E. M. Shikotra A. Singapuri A. Sereno M. . (2021). Physical, cognitive, and mental health impacts of COVID-19 after hospitalisation (PHOSP-COVID): a UK multicentre, prospective cohort study. Lancet Respir. Med. 9, 1275–1287. doi: 10.1016/S2213-2600(21)00383-0, 34627560 PMC8497028

[ref16] Faustino B. Lopes P. Oliveira J. Campaioli G. Rondinone M. Bomfim H. . (2019). Psychometric and rash analysis of the UCLA loneliness scale-16 in a Portuguese sample of older adults. Psychol. Stud. 64, 140–146. doi: 10.1007/s12646-019-00483-5

[ref17] Ferreira P. L. Ferreira L. N. Pereira L. N. (2012). Medidas sumário física e mental de estado de saúde para a população portuguesa. (Physical and mental summary measures of health state for the portuguese population). Rev. Port. Saude Publica 30, 163–171. doi: 10.1016/j.rpsp.2012.12.007

[ref18] Fillion S. S. (2024). Posttraumatic growth during COVID19 in students: the roles of coping, trait emotional intelligence, and perceived social support (master’s thesis). Trent University, Peterborough (ON). Available at: ProQuest Dissertations & Theses Global

[ref19] Furman D. Campisi J. Verdin E. Carrera-Bastos P. Targ S. Franceschi C. . (2019). Chronic inflammation in the etiology of disease across the life span. Nat. Med. 25, 1822–1832. doi: 10.1038/s41591-019-0675-0, 31806905 PMC7147972

[ref20] Gabarrell-Pascuet A. Koyanagi A. Felez-Nobrega M. Cristóbal-Narváez P. Mortier P. Vilagut G. . (2023). The association of age with depression, anxiety, and posttraumatic stress symptoms during the COVID-19 pandemic in Spain: the role of loneliness and prepandemic mental disorder. Psychosom. Med. 85, 42–52. doi: 10.1097/PSY.0000000000001146, 36201774

[ref21] Garrigues E. Janvier P. Kherabi Y. Le Bot A. Hamon A. Gouze H. . (2020). Post-discharge persistent symptoms and health-related quality of life after hospitalization for COVID-19. J. Infect. 81, e4–e6. doi: 10.1016/j.jinf.2020.08.029, 32853602 PMC7445491

[ref22] Gaskill P. J. Khoshbouei H. (2022). Dopamine and norepinephrine are embracing their immune side and so should we. Curr. Opin. Neurobiol. 77:102626. doi: 10.1016/j.conb.2022.102626, 36058009 PMC10481402

[ref23] Glazebrook A. Shakespeare-Finch J. Andrew B. van der Meer J. (2023). Posttraumatic growth EEG neuromarkers: translational neural comparisons with resilience and PTSD in trauma-exposed healthy adults. Eur. J. Psychotraumatol. 14:2272477. doi: 10.1080/20008066.2023.2272477, 37965734 PMC10653763

[ref24] Hair J. F. Black W. C. Babin B. J. Anderson R. E. (2010). Multivariate data analysis. 7th Edn. Upper Saddle River, NJ: Pearson.

[ref25] Hamer M. Chida Y. (2011). Life satisfaction and inflammatory biomarkers: the 2008 Scottish health survey 1. Jpn. Psychol. Res. 53, 133–139. doi: 10.1111/j.1468-5884.2011.00460.x

[ref26] Han Q. Zheng B. Daines L. Sheikh A. (2022). Long-term sequelae of COVID-19: a systematic review and meta-analysis of one-year follow-up studies on post-COVID symptoms. Pathogens 11:269. doi: 10.3390/pathogens11020269, 35215212 PMC8875269

[ref27] Hariyanto T. I. Japar K. V. Kwenandar F. Damay V. Siregar J. I. Lugito N. P. H. . (2021). Inflammatory and hematologic markers as predictors of severe outcomes in COVID-19 infection: a systematic review and meta-analysis. Am. J. Emerg. Med. 41, 110–119. doi: 10.1016/j.ajem.2020.12.076, 33418211 PMC7831442

[ref28] Harsanyi S. Kupcova I. Danisovic L. Klein M. (2023). Selected biomarkers of depression: what are the effects of cytokines and inflammation? Int. J. Mol. Sci. 24:578. doi: 10.3390/ijms24010578, 36614020 PMC9820159

[ref29] Hassamal S. (2023). Chronic stress, neuroinflammation, and depression: an overview of pathophysiological mechanisms and emerging anti-inflammatories. Front. Psych. 14:1130989. doi: 10.3389/fpsyt.2023.1130989, 37252156 PMC10213648

[ref30] Hayes A. F. (2018). Introduction to mediation, moderation, and conditional process analysis: a regression-based approach. 2nd Edn. New York, NY: Guilford Press.

[ref31] Huang C. Huang L. Wang Y. Li X. Ren L. Gu X. . (2023). 6-month consequences of COVID-19 in patients discharged from hospital: a cohort study. Lancet 401, 21–33. doi: 10.1016/S0140-6736(23)00810-3 PMC10258565 37321233

[ref32] Huang L. Yao Q. Gu X. Wang Q. Ren L. Wang Y. . (2021). 1-year outcomes in hospital survivors with COVID-19: a longitudinal cohort study. Lancet 398, 747–758. doi: 10.1016/S0140-6736(21)01755-4, 34454673 PMC8389999

[ref33] Illiano P. Leo D. Gainetdinov R. R. Pardo M. (2021). Early adolescence prefrontal cortex alterations in female rats lacking dopamine transporter. Biomedicine 9:157. doi: 10.3390/biomedicines9020157, 33562738 PMC7914429

[ref1101] Juczyński Z. Kwiecińska L. Ogińska-Bulik J. (2023). Ruminations as predictors of post-traumatic stress disorder after hospitalization for Covid-19. Ruminacje jako wyznaczniki zespołu stresu pourazowego po hospitalizacji z powodu Covid-19. Psychiatr. Pol. 57, 1011–1022. doi: 10.12740/PP/152817, 38345125

[ref34] Kachadourian L. K. Harpaz-Rotem I. Tsai J. Southwick S. Pietrzak R. H. (2021). Mindfulness as a mediator between trauma exposure and mental health outcomes: results from the National Health and resilience in veterans study. Psychol. Trauma Theory Res. Pract. Policy 13, 223–230. doi: 10.1037/tra0000995, 33475404 PMC8500672

[ref35] Kalaiselvan P. Yingchoncharoen P. Thongpiya J. Motes A. Nugent K. (2023). COVID-19 infections and inflammatory markers in patients hospitalized during the first year of the pandemic. J. Prim. Care Community Health 14:21501319231206911. doi: 10.1177/21501319231206911, 37864436 PMC10590050

[ref36] Khraisat B. Toubasi A. AlZoubi L. Al-Sayegh T. Mansour A. (2022). Meta-analysis of prevalence: the psychological sequelae among COVID-19 survivors. Int. J. Psychiatry Clin. Pract. 26, 234–243. doi: 10.1080/13651501.2021.1993924, 34709105

[ref37] Kim N. H. Kim S. H. Hyun S. Y. Kang D. R. Oh M. J. Kim D. (2018). Mediating role of anxiety and depression in the relationship between posttraumatic stress symptoms and illness intrusiveness. J. Korean Med. Sci. 33:e284. doi: 10.3346/jkms.2018.33.e284, 30402049 PMC6209764

[ref38] Kline R. B. (2015). Principles and practice of structural equation modeling. 4th Edn. New York, NY: Guilford Press.

[ref39] Lamela D. Figueiredo B. Bastos A. Martins H. (2013). Posttraumatic growth inventory short form—Portuguese version (Portuguese PTGI-SF, PTGI-SF) [database record]. APA PsycTests. doi: 10.1037/t28139-000

[ref40] Lauzon N. M. Ahmad T. Laviolette S. R. (2012). Dopamine D4 receptor transmission in the prefrontal cortex controls the salience of emotional memory via modulation of calcium calmodulin-dependent kinase II. Cereb. Cortex 22, 2486–2494. doi: 10.1093/cercor/bhr326, 22120417 PMC4705337

[ref41] Lauzon N. M. Bechard M. Ahmad T. Laviolette S. R. (2013). Supra-normal stimulation of dopamine D1 receptors in the prelimbic cortex blocks behavioral expression of both aversive and rewarding associative memories through a cyclic-AMP-dependent signaling pathway. Neuropharmacology 67, 104–114. doi: 10.1016/j.neuropharm.2012.10.029, 23164618

[ref42] Li S. Shu H. Wu Y. Li F. Yang J. Luo L. . (2025). Post-traumatic growth promotes resilience development: a longitudinal mediation model. J. Affect. Disord. 368, 727–733. doi: 10.1016/j.jad.2024.09.113, 39299589

[ref43] Limanaqi F. Zecchini S. Dino B. Strizzi S. Cappelletti G. Utyro O. . (2022). Dopamine reduces SARS-CoV-2 replication *in vitro* through downregulation of D2 receptors and upregulation of type-I interferons. Cells 11:1691. doi: 10.3390/cells11101691, 35626728 PMC9139638

[ref44] Liu J. Wei S. Qiu G. Li N. Wang D. Wu X. . (2023). Relationship between rumination and post-traumatic growth in mobile cabin hospital nurses: the mediating role of psychological resilience. Prev. Med. Rep. 34:102266. doi: 10.1016/j.pmedr.2023.102266, 37288138 PMC10241969

[ref45] Liu M. N. Tian X. Y. Fang T. Wu N. Li H. Li J. (2023). Insights into the involvement and therapeutic target potential of the dopamine system in posttraumatic stress disorder. Mol. Neurobiol. 60, 3708–3723. doi: 10.1007/s12035-023-03312-z 36933147

[ref47] Liu Y. Du X. Chen J. Jin Y. Peng L. Wang H. H. X. . (2020). Neutrophil-to-lymphocyte ratio as an independent risk factor for mortality in hospitalized patients with COVID-19. J. Infect. 81, e6–e12. doi: 10.1016/j.jinf.2020.04.002, 32283162 PMC7195072

[ref48] Lopes A. Rocha J. (2013). Convergent validity of impact of event scale-revised and impact of event scale-6 Portuguese versions. (master’s thesis). Instituto Superior de Ciências da Saúde–Norte (CESPU), Gandra (Portugal).

[ref49] Maamar M. Artime A. Pariente E. Fierro P. Ruiz Y. Gutiérrez S. . (2022). Post-COVID-19 syndrome, low-grade inflammation and inflammatory markers: a cross-sectional study. Curr. Med. Res. Opin. 38, 901–909. doi: 10.1080/03007995.2022.2042991, 35166141 PMC8935459

[ref50] Martins V. Serrão C. Teixeira A. Castro L. Duarte I. (2022). The mediating role of life satisfaction in the relationship between depression, anxiety, stress and burnout among Portuguese nurses during COVID-19 pandemic. BMC Nurs. 21:188. doi: 10.1186/s12912-022-00958-3, 35850892 PMC9289090

[ref51] Mazza M. G. De Lorenzo R. Conte C. Poletti S. Vai B. Bollettini I. . (2020). Anxiety and depression in COVID-19 survivors: role of inflammatory and clinical predictors. Brain Behav. Immun. 89, 594–600. doi: 10.1016/j.bbi.2020.07.037, 32738287 PMC7390748

[ref52] McKenna F. McLaughlin P. J. Lewis B. J. Sibbring G. C. Cummerson J. A. Bowen-Jones D. . (2002). Dopamine receptor expression on human T- and B-lymphocytes, monocytes, neutrophils, eosinophils and NK cells: a flow cytometric study. J. Neuroimmunol. 132, 34–40. doi: 10.1016/s0165-5728(02)00280-1, 12417431

[ref53] Michopoulos V. Powers A. Gillespie C. F. Ressler K. J. Jovanovic T. (2017). Inflammation in fear- and anxiety-based disorders: PTSD, GAD, and beyond. Neuropsychopharmacology 42, 254–270. doi: 10.1038/npp.2016.146, 27510423 PMC5143487

[ref54] Müller K. Poppele I. Ottiger M. Weber R. C. Stegbauer M. Schlesinger T. (2025). Course of neuropsychological health in post-COVID patients differs 6 and 12 months after inpatient rehabilitation. Front. Psych. 16:1460097. doi: 10.3389/fpsyt.2025.1460097, 40352374 PMC12062137

[ref55] National Institute for Health and Care Excellence. (2020). COVID-19 rapid guideline: managing the long-term effects of COVID-19 (NICE guideline no. NG188). Available online at: https://www.nice.org.uk/guidance/ng188 (Accessed June 16, 2020).

[ref56] Nowakowski A. C. Graves K. Y. Sumerau J. E. (2016). Mediation analysis of relationships between chronic inflammation and quality of life in older adults. Health Qual. Life Outcomes 14:46. doi: 10.1186/s12955-016-0452-4, 27001461 PMC4802844

[ref57] O’Connor D. B. Wilding S. Ferguson E. Cleare S. Wetherall K. McClelland H. . (2022). Effects of COVID-19-related worry and rumination on mental health and loneliness during the pandemic: longitudinal analyses of adults in the UK COVID-19 mental health & wellbeing study. J. Ment. Health 32, 1122–1133. doi: 10.1080/09638237.2022.2069716, 35579054

[ref58] Özgüç S. Tanrıverdi D. Güner M. Kaplan S. N. (2022). The examination of stress symptoms and posttraumatic growth in the patients diagnosed with COVID-19. Intensive Crit. Care Nurs. 73:103274. doi: 10.1016/j.iccn.2022.103274 35729040 PMC9159976

[ref59] Pacheco R. Contreras F. Zouali M. (2014). The dopaminergic system in autoimmune diseases. Front. Immunol. 5:117. doi: 10.3389/fimmu.2014.00117, 24711809 PMC3968755

[ref60] Pais-Ribeiro J. Silva I. Ferreira T. Martins A. Meneses R. Baltar M. (2007). Validation study of a Portuguese version of the hospital anxiety and depression scale. Psychol. Health Med. 12, 225–237. doi: 10.1080/13548500500524088, 17365902

[ref61] Pansarasa O. Garofalo M. Scarian E. Dragoni F. Garau J. Di Gerlando R. . (2022). Biomarkers in human peripheral blood mononuclear cells: the state of the art in amyotrophic lateral sclerosis. Int. J. Mol. Sci. 23:2580. doi: 10.3390/ijms23052580, 35269723 PMC8910056

[ref62] Penninx B. W. J. H. Benros M. E. Klein R. S. Vinkers C. H. (2022). How COVID-19 shaped mental health: from infection to pandemic effects. Nat. Med. 28, 2027–2037. doi: 10.1038/s41591-022-02028-2, 36192553 PMC9711928

[ref63] Pitron V. Cantenys W. Herbelin A. Bottemanne H. Dzierzynski N. Caumes E. . (2022). Factors associated with posttraumatic stress symptoms 3 and 6 months after hospitalization for COVID-19: a longitudinal multicenter study. J. Clin. Psychiatry 84:21m14277. doi: 10.4088/JCP.21m14277, 36479951

[ref64] Rahmati M. Udeh R. Kang J. Dolja-Gore X. McEvoy M. Kazemi A. . (2025). Long-term sequelae of COVID-19: a systematic review and meta-analysis of symptoms 3 years post-SARS-CoV-2 infection. J. Med. Virol. 97:e70429. doi: 10.1002/jmv.70429, 40476637 PMC12143191

[ref65] Rahmoune H. Guest P. C. (2018). “Studies of isolated peripheral blood cells as a model of immune dysfunction” in Investigations of early nutrition effects on long-term health: methods and applications. ed. Guest P. C. (Cham: Springer), 221–229. 10.1007/978-1-4939-7614-0_12 29380315

[ref66] Ramos C. Figueiras L. Lopes M. Leal I. Tedeschi R. (2015). Inventário de ruminação relacionada com o acontecimento: qualidades psicométricas na população portuguesa. (Event-related rumination inventory: psychometric properties on a Portuguese sample). Psic. Saúde Doenças 16, 299–310. doi: 10.15309/15psd160303

[ref67] Rasmi Y. Shokati A. Hatamkhani S. Farnamian Y. Naderi R. Jalali L. (2024). Assessment of the relationship between the dopaminergic pathway and severe acute respiratory syndrome coronavirus 2 infection, with related neuropathological features, and potential therapeutic approaches in COVID-19 infection. Rev. Med. Virol. 34:e2506. doi: 10.1002/rmv.2506, 38282395

[ref68] Renna M. E. (2021). A review and novel theoretical model of how negative emotions influence inflammation: the critical role of emotion regulation. Brain Behav. Immun. Health 18:100397. doi: 10.1016/j.bbih.2021.100397, 34927103 PMC8649080

[ref69] Reppold C. Kaiser V. Zanon C. Hutz C. Casanova J. R. Almeida L. S. (2019). Escala de Satisfação com a Vida: Evidências de validade e precisão junto de universitários portugueses. (Satisfaction with Life Scale: Evidences of validity and reliability among portuguese college students). Rev. Estud. Investig. Psicol. Educ. 6, 15–23. doi: 10.17979/reipe.2019.6.1.4617

[ref70] Roncati L. Galeazzi C. Bartolacelli G. Caramaschi S. (2024). A real-world nationwide study on COVID-19 trend in Italy during the autumn–winter season of 2020 (before mass vaccination) and 2021 (after mass vaccination) integrated with a retrospective analysis of the mortality burden per year. Microorganisms 12:435. doi: 10.3390/microorganisms12030435, 38543486 PMC10972431

[ref71] Saponjic J. Mejías R. Nikolovski N. Dragic M. Canak A. Papoutsopoulou S. . (2024). Experimental models to study immune dysfunction in the pathogenesis of Parkinson’s disease. Int. J. Mol. Sci. 25:4330. doi: 10.3390/ijms25084330, 38673915 PMC11050170

[ref72] Sarkar C. Basu B. Chakroborty D. Dasgupta P. S. Basu S. (2010). The immunoregulatory role of dopamine: an update. Brain Behav. Immun. 24, 525–528. doi: 10.1016/j.bbi.2009.10.015, 19896530 PMC2856781

[ref73] Sipowicz K. Pietras T. Mosiołek A. Sobstyl M. Ring M. Kamecki K. . (2023). The sense of loneliness and meaning in life in post-COVID convalescents-a preliminary study. Front. Psych. 14:1296385. doi: 10.3389/fpsyt.2023.1296385, 38188044 PMC10768000

[ref74] Soper D. S. (2019). A-priori sample size calculator for hierarchical multiple regression [computer software]. Available online at: http://www.danielsoper.com/statcalc (Accessed June 16, 2019).

[ref75] Sun N. Wei L. Wang H. Wang X. Gao M. Hu X. . (2021). Qualitative study of the psychological experience of COVID-19 patients during hospitalization. J. Affect. Disord. 278, 15–22. doi: 10.1016/j.jad.2020.08.040, 32949869 PMC7444461

[ref76] Szabo Y. Z. Burns C. M. Lantrip C. (2022). Understanding associations between rumination and inflammation: a scoping review. Neurosci. Biobehav. Rev. 135:104523. doi: 10.1016/j.neubiorev.2022.104523, 34998832 PMC8957598

[ref77] Tedeschi R. G. Calhoun L. G. (2004). Posttraumatic growth: conceptual foundations and empirical evidence. Psychol. Inq. 15, 1–18. doi: 10.1207/s15327965pli1501_01

[ref78] Thompson E. J. Stafford J. Moltrecht B. Huggins C. F. Kwong A. S. F. Shaw R. J. . (2022). Psychological distress, depression, anxiety, and life satisfaction following COVID-19 infection: evidence from 11 UK longitudinal population studies. Lancet Psychiatry 9, 894–906. doi: 10.1016/S2215-0366(22)00307-8, 36244359 PMC9560745

[ref79] Thye A. Y. Law J. W. Tan L. T. Pusparajah P. Ser H. L. Thurairajasingam S. . (2022). Psychological symptoms in COVID-19 patients: insights into pathophysiology and risk factors of long COVID-19. Biol. 11:61. doi: 10.3390/biology11010061, 35053059 PMC8773222

[ref80] Tokano M. Takagi R. Kawano M. Maesaki S. Tarumoto N. Matsushita S. (2022). Signaling via dopamine and adenosine receptors modulate viral peptide-specific and T-cell IL-8 response in COVID-19. Immunol. Med. 45, 162–167. doi: 10.1080/25785826.2022.2079369, 35623041

[ref81] Torrisi S. A. Leggio G. M. Drago F. Salomone S. (2019). Therapeutic challenges of post-traumatic stress disorder: focus on the dopaminergic system. Front. Pharmacol. 10:404. doi: 10.3389/fphar.2019.00404, 31057408 PMC6478703

[ref82] Uchino B. N. de Grey R. G. K. Cronan S. Smith T. W. Diener E. Joel S. . (2018). Life satisfaction and inflammation in couples: an actor-partner analysis. J. Behav. Med. 41, 22–30. doi: 10.1007/s10865-017-9880-9, 28884245 PMC5766426

[ref83] Ulloque-Badaracco J. R. Ivan Salas-Tello W. Al-kassab-Córdova A. Alarcón-Braga E. A. Benites-Zapata V. A. Maguiña J. L. . (2021). Prognostic value of neutrophil-to-lymphocyte ratio in COVID-19 patients: a systematic review and meta-analysis. Int. J. Clin. Pract. 75:e14596. doi: 10.1111/ijcp.14596, 34228867 PMC9614707

[ref84] Vlake J. H. Wesselius S. Genderen M. E. Bommel J. de Boxma-Klerk B. Wils E. J., 2021 Psychological distress and health-related quality of life in patients after hospitalization during the COVID-19 pandemic: a single-center, observational study PLoS One 16:e0255774 doi: 10.1371/journal.pone.0255774 34379644 PMC8357130

[ref85] Wang T. Nowrangi D. Yu L. Lu T. Tang J. Han B. . (2018). Activation of dopamine D1 receptor decreased NLRP3-mediated inflammation in intracerebral hemorrhage mice. J. Neuroinflammation 15:2. doi: 10.1186/s12974-017-1039-7, 29301581 PMC5753458

[ref86] World Health Organization. (2024). COVID-19 epidemiological update—24 December 2024. Available online at: https://cdn.who.int/media/docs/default-source/documents/emergencies/20241224_covid-19_epi_update_special-edition.pdf?sfvrsn=b0a6ddaf_1&download=true (accessed August 11, 2025)

[ref87] Yan Y. Jiang W. Liu L. Wang X. Ding C. Tian Z. . (2015). Dopamine controls systemic inflammation through inhibition of NLRP3 inflammasome. Cell 160, 62–73. doi: 10.1016/j.cell.2014.11.047, 25594175

[ref88] Yang J. J. Jiang W. (2020). Immune biomarker alterations in post-traumatic stress disorder: a systematic review and meta-analysis. J. Affect. Disord. 268, 39–46. doi: 10.1016/j.jad.2020.02.044 32158005

[ref89] Yang X. Hou C. Shen Y. Zhang M. Zhang K. Wang F. . (2022). Two-year health outcomes in hospitalized COVID-19 survivors in China. JAMA Netw. Open 5:e2231790. doi: 10.1001/jamanetworkopen.2022.31790, 36107425 PMC9478774

[ref90] Yontar G. Mutlu E. A. (2024). Neutrophil-to-lymphocyte, platelet-to-lymphocyte ratios and systemic immune-inflammation index in patients with post-traumatic stress disorder. BMC Psychiatry 24:966. doi: 10.1186/s12888-024-06439-y, 39741243 PMC11686920

[ref91] Zaffaroni M. Marino F. Bombelli R. Rasini E. Monti M. Ferrari M. . (2008). Therapy with interferon-β modulates endogenous catecholamines in lymphocytes of patients with multiple sclerosis. Exp. Neurol. 214, 315–321. doi: 10.1016/j.expneurol.2008.08.015 18824168

[ref92] Zagaria A. Fiori V. Vacca M. Lombardo C. Pariante C. M. Ballesio A. (2024). Inflammation as a mediator between adverse childhood experiences and adult depression: a meta-analytic structural equation model. J. Affect. Disord. 357, 85–96. doi: 10.1016/j.jad.2024.04.072, 38677656

[ref93] Zhou P. Deng M. Wu J. Lan Q. Yang H. Zhang C. (2021). Ventral tegmental area dysfunction and disruption of dopaminergic homeostasis: implications for post-traumatic stress disorder. Mol. Neurobiol. 58, 2423–2434. doi: 10.1007/s12035-020-02278-6, 33428093

[ref94] Zilioli S. Jiang Y. (2021). Endocrine and immunomodulatory effects of social isolation and loneliness across adulthood. Psychoneuroendocrinology 128:105194. doi: 10.1016/j.psyneuen.2021.105194, 33932766

[ref95] Zuschlag Z. D. Compean E. Nietert P. Lauzon S. Hamner M. Wang Z. (2021). Dopamine transporter (DAT1) gene in combat veterans with PTSD: a case-control study. Psychiatry Res. 298:113801. doi: 10.1016/j.psychres.2021.113801, 33636518 PMC8182484

