# Supplementary material for: Quality of life in hospitalized COVID-19 patients: the role of psychosocial, inflammatory, and dopaminergic pathways
Source: Front Psychol. 2026 Jan 7;16:1684510. doi: 10.3389/fpsyg.2025.1684510 (PMC12819234; doi:10.3389/fpsyg.2025.1684510)
Supplement: Supplementary file 1 [file Table_1.DOCX]

**Supplementary material S1**

Real-Time PCR for DR (DRD1–DRD5) Expression in PBMCs

Previously isolated PBMCs, stored at –80 °C, were thawed and resuspended in medium to remove cryoprotectants. After centrifugation, pellets were stabilized with RNAlater™ (Thermo Fisher Scientific). Total RNA was purified using the GeneJET RNA Purification Kit with DNase treatment, and RNA integrity was confirmed (RIN 8–9). Complementary DNA (cDNA) was synthesized using the High-Capacity cDNA Reverse Transcription Kit, with negative controls to confirm absence of genomic DNA. Expression levels of DRD1–DRD5 were quantified by real-time PCR using a dual-chemistry strategy: SYBR Green for DRD1 and DRD4, and probe-based assays for DRD2, DRD3, and DRD5. SYBR Green chemistry was adopted for DRD1 and DRD4 based on well-established primer specificity and cost-efficiency, whereas TaqMan probe-based assays were used for DRD2, DRD3, and DRD5 due to higher sequence homology requiring increased specificity. Assay efficiency, specificity, and performance were harmonized through standard curve validation (97–103% efficiency, R² > 0.99), melt-curve analysis for SYBR reactions, and normalization to a stable housekeeping gene. This ensured full comparability of quantitative expression data across chemistries. A VIC-labeled housekeeping gene was co-amplified for normalization. All assays demonstrated high specificity and efficiency (97–103%). Gene expression was analyzed by the comparative ΔΔCt method, with relative expression reported as 2^–ΔΔCt, normalized to the housekeeping gene and a calibrator sample. Replicates with variance > 0.3 Ct were excluded. Details of assay design are provided in Supplementary Table S1.

| **Table 1A**  *Real-Time PCR gene expression.Data are from RefSeq - NCBI Reference Sequence Database (<http://www.ncbi.nlm.nih.gov/refseq/>)* | | | | | | |
| --- | --- | --- | --- | --- | --- | --- |
| Gene | UniGene ID | Interrogated Sequence | Translated Protein | Exon Boundary | Assay Location | Amplicon Length |
| DRD_1_ | Hs.2624 | [NM_000794.3](http://www.ncbi.nlm.nih.gov/entrez/viewer.fcgi?val=NM_000794.3) | [NP_000785.1](http://www.ncbi.nlm.nih.gov/entrez/viewer.fcgi?val=NP_000785.1) | 1-2 | 462-1620 | 110 |
| DRD_2_ | Hs.73893 | [NM_000795.3](http://www.ncbi.nlm.nih.gov/entrez/viewer.fcgi?val=NM_000795.3) | [NP_000786.1](http://www.ncbi.nlm.nih.gov/entrez/viewer.fcgi?val=NP_000786.1) | 2-3 | 524 | 64 |
| DRD_3_ | Hs.121478 | [NM_033663.3](http://www.ncbi.nlm.nih.gov/entrez/viewer.fcgi?val=NM_033663.3) | [NP_387512.3](http://www.ncbi.nlm.nih.gov/entrez/viewer.fcgi?val=NP_387512.3) | 3-4 | 809-725 | 73 |
| DRD_4_ | Hs.99922 | [NM_000797.3](http://www.ncbi.nlm.nih.gov/entrez/viewer.fcgi?val=NM_000797.3) | [NP_000788.2](http://www.ncbi.nlm.nih.gov/entrez/viewer.fcgi?val=NP_000788.2) | 1-2 | 285-283 | 99 |
| DRD_5_ | Hs.380681 | [NM_000798.4](http://www.ncbi.nlm.nih.gov/entrez/viewer.fcgi?val=NM_000798.4) | [NP_000789.1](http://www.ncbi.nlm.nih.gov/entrez/viewer.fcgi?val=NP_000789.1) | 1-1 | 1092-744 | 88 |
| 18SrRNA | X03205.1 | N/A | N/A | N/A | N/A | 187 |
| *Note.* DRD_1_ = dopaminergic receptor type 1; DRD_2_ = dopaminergic receptor type 2; DRD_3_ = dopaminergic receptor type 3; DRD_4_ = dopaminergic receptor type 4; DRD_5_ = dopaminergic receptor type 5. | | | | | | |
